# TNF receptor-associated factor 5 gene confers genetic predisposition to acute anterior uveitis and pediatric uveitis

**DOI:** 10.1186/ar4293

**Published:** 2013-09-11

**Authors:** Qin Xiang, Lu Chen, Jing Fang, Shengping Hou, Lin Wei, Lin Bai, Yunjia Liu, Yan Zhou, Aize Kijlstra, Peizeng Yang

**Affiliations:** 1 The First Affiliated Hospital of Chongqing Medical University, Chongqing Key Laboratory of Ophthalmology and Chongqing Eye Institute, Chongqing 400016, PR China; 2 University Eye Clinic Maastricht, Maastricht, The Netherlands

## Abstract

**Introduction:**

TNF Receptor-Associated Factor 5 (*TRAF5*) has been shown to be associated with autoimmune disease. The current study sought to investigate the potential association of *TRAF5* with acute anterior uveitis (AAU) and pediatric uveitis in Han Chinese.

**Methods:**

Three *TRAF5* SNPs were analyzed in 450 AAU patients with or without ankylosing spondylitis (AS), 458 pediatric uveitis patients, and 1,601 healthy controls by using polymerase chain reaction-restriction fragment length polymorphism (PCR-RFLP) or TaqMan SNP Genotyping Assay. Numerous variables were evaluated, including age, sex distribution, and clinical and laboratory observations.

**Results:**

Two SNPs (rs6540679, rs12569232) of *TRAF5* were associated with pediatric uveitis, and rs12569232 also showed a relation with the presence of microvascular leakage. No significant associations were found when patients were subdivided according to their rheumatoid factor (RF) or anti-nuclear antibody (ANA) status or whether they had juvenile idiopathic arthritis (JIA). Rs12569232 predisposed to AAU and its subgroups (with ankylosing spondylitis (AS) or HLA-B27 positive). No association was found between rs10863888 and either pediatric uveitis or AAU.

**Conclusion:**

This study revealed that *TRAF5* is involved in the development of AAU and pediatric uveitis. Further stratified analysis according to the clinical and laboratory observations suggested that rs12569232/*TRAF5* may play a role in the development of retinal vasculitis.

## Introduction

Uveitis is a manifestation of complex processes and a major etiologic factor of visual loss. Uveitis can be categorized into several phenotypes. Among them, acute anterior uveitis (AAU) is the most common entity encountered [1]. Many patients with AAU are HLA-B27 positive, and a strong association with ankylosing spondylitis (AS) is observed in this subgroup of patients [2,3]. Similarly, 30% to 50% of patients with HLA-B27-positive AS will have an episode of AAU in the course of their disease [4-6]. Both heredity and environmental factors, especially bacterial triggers, are thought to be involved in the development of AAU and AS [7-9]. Half of the AAU cases are HLA-B27 positive [8], and the association of HLA-B27 with AS is found in almost all ethnic groups [10-13].

Pediatric uveitis is one of the most common reasons for childhood blindness. Children with uveitis comprise 2.2% to 10.6% of the total number of uveitis patients [14,15], and anterior uveitis is the most frequent type of involvement in the majority of cases [16,17]. About 60% of pediatric uveitis cases are diagnosed as idiopathic uveitis, followed by juvenile idiopathic arthritis (JIA)-associated anterior uveitis [17]. Rheumatoid factor (RF), anti-nuclear antibody (ANA), and fundus fluorescein angiography (FFA) are regarded as important tests for pediatric uveitis and JIA.

Recent years witnessed a rapid improvement in the understanding of the genetic basis of AS, AAU and pediatric uveitis, including more than 69 subtypes of HLA-B27, as well as other immune-response genes, such as *IL-1A, ERAP1, IL-23R* in AS [18], *TLR2, TLR4, TNF* in AAU [19,20], and chemokine and chemokine-receptor genes in pediatric uveitis [21]. The involvement of these latter genes is, however, not consistent among the various ethnic groups throughout the world, as evidenced by association studies concerning *IL-23R* in AS [22-24], and further studies in well-defined ethnic populations are needed to understand how non-HLA-B27 genes can contribute to the development of autoimmune or autoinflammatory diseases.

TNF receptor-associated factor 5 (*TRAF5*) is a signal transducer that links members of the tumor necrosis factor (TNF)-receptor family to different signaling pathways, regulating NF-κB and probably JNK activation [25-27]. NF-κB is considered a common transcription factor that is critical for innate and adaptive immunity and has been implied to play a role in autoimmune and autoinflammatory diseases, such as JIA [28], rheumatoid arthritis [29], and AS [30]. Inhibition of NF-κB has been verified as a treatment for AAU, experimental autoimmune encephalomyelitis, experimental autoimmune uveitis and inflammatory arthritis [31-34]. Recent studies have shown that *TRAF5* polymorphisms are associated with rheumatoid arthritis (RA) [35]. As mentioned earlier, uveitis and arthritis have overlapping manifestations, and because the relation between *TRAF5* with uveitis has not yet been reported, we decided to investigate the role of *TRAF5* polymorphisms in two forms of uveitis, AAU and pediatric uveitis.

## Methods

### Subjects

The study included 450 AAU patients, 458 pediatric uveitis patients, and 1,601 healthy adult controls. All patients and controls were Han Chinese, as confirmed by their ID cards. The diagnosis of AS was based on the modified New York criteria [36], and AAU patients were diagnosed principally according to clinical manifestations [37,38]. Pediatric uveitis was defined as uveitis first seen in patients younger than 16 years. Children with Behḉet disease, Vogt-Koyanagi-Harada syndrome, or definite infectious uveitis entities were excluded from the study. JIA was defined as arthritis of at least 6 weeks’ duration without any other identifiable cause in children younger than 16 years. Subjects were excluded if they had Behḉet disease, Vogt-Koyanagi-Harada syndrome, or another type of uveitis. Blood samples of the patients and controls were obtained from the Uveitis Study Center of the Sun Yat-sen University (Guangzhou, P.R. China) and the First Affiliated Hospital of Chongqing Medical University (Chongqing, P.R. China) between 2005 and 2013. All the subjects participating in our study came from all over the country, as evidenced by their ID cards. The study was approved by the Local Ethics Research Committee of The First Affiliated Hospital of Chongqing Medical University, Chongqing, China (Permit Number: 2009–201008), and all tested subjects gave their written informed consent; for children, the informed consent was obtained from their parents or guardians. The procedures followed the tenets of the Declaration of Helsinki.

### Clinical and laboratory observations

Pediatric uveitis patients underwent a uveitis screening protocol encompassing ANA (*n* = 176; positive rate was 17.0%), RF (*n* = 187; positive rate was 15.5%), and FFA (*n* = 271; microvascular leakage rate detected by FFA was found in 63.5% of tested patients). An ANA titer above 1:100 and an RF level above 20.0 IU/ml were considered positive. FFA was considered a positive result when dye leakage from a retinal vessel was observed. Of the AAU patients, 209 had AS, and of the 332 subjects tested for HLA-B27, 267 (80.4%) were positive. All these tests were performed in the First Affiliated Hospital of Chongqing Medical University (Chongqing, P.R. China). The clinical characteristics of the patients are shown in Tables 1 and 2.

**Table 1:** Clinical features of the investigated AAU patients

**Clinical features**	***n *** **(total = 450)**	**%**
Age (years)	39.2 ± 10.0	
Male	314	69.8
Female	136	30.2
Uveitis	450	100
AAU patients with AS	209	46.4
AAU patients without AS	241	53.6
HLA-B27(+)	267 (332 tested)	80.4
HLA-B27(-)	65 (332 tested)	19.6

AAU, acute anterior uveitis; AS, ankylosing spondylitis. +, having this feature; -, not having this feature.

**Table 2:** Clinical features of the investigated pediatric uveitis patients

**Clinical features**	**Pediatric uveitis**	
	***n *** **(total = 458)**	**%**
Age at onset (years ± SD)	9.0 ± 4	
Male	214	46.7
Female	244	53.3
Uveitis	458	100
Pediatric uveitis with JIA	60	13.1
FFA(+)	172 (271 tested)	63.5
ANA(+)	30 (176 tested)	17.0
RF(+)	29b (187 tested)	15.5

JIA, juvenile idiopathic arthritis; FFA, fundus fluorescein angiography; ANA, anti-nuclear antigen; RF, rheumatoid factor. +, having this feature; -, not having this feature.

### SNP selection, DNA extraction, and genotyping

The polymorphisms used in this study were based on earlier reports in RA [35], including rs6540679, rs7514863, rs12569232, and rs10863888. Genomic DNA samples of patients and healthy controls were extracted by using the QIAamp DNA Blood Mini Kit (Qiagen, Valencia, CA, USA) and genotyped by polymerase chain reaction-restriction fragment length polymorphism (PCR-RFLP) for rs6540679 or by TaqMan SNP Genotyping Assay for rs12569232 (TaqMan assay ID: C_26176827_20) and rs10863888 (TaqMan assay ID: C_26176858_10) using the ABI 7500 Real-Time PCR system. Digestion PCR products were visualized on a 4% agarose gel and stained with GoldViewTM (SBS Genetech, Beijing, China). Unfortunately, as we designed six PCR primers for rs7514863, none of their PCR product was satisfactory to go to the digestion step. No TaqMan SNP Genotyping Assay for it is available, we had to give up this SNP, although it was found to have been significantly associated with RA.

All the tests were performed according to the manufacturers’ instructions. Direct sequencing was also performed by the Beijing Biomed Co., Ltd (Beijing, China), by using randomly selected subjects (10% of all samples) to validate the method used in this study.

### Statistical analysis

Test of Hardy-Weinberg equilibrium (HWE), evaluation of genotype and allele frequencies, and calculation of odds ratios (ORs) and 95% confidence intervals (95% CIs) were done by following the method of Chen *et al*. [39]. The *P* values were corrected (*P*_c_) by using the Bonferroni correction method. *P*_c_ < 0.05 was considered significant. The linkage disequilibriums (LDs) of these tested SNPs were compared by using Haploview v3.32.

## Results

### *TRAF5* gene implicated in susceptibility to acute anterior uveitis

The three SNPs (rs6540679, rs12569232, rs10863888) of *TRAF5* were successfully genotyped and conformed to Hardy-Weinberg expectation in controls. The chosen SNPs were not in linkage disequilibrium with each other.

The frequencies of the CG genotype and C allele of rs12569232 in the AAU group were much lower than those in the healthy controls (*P*_c_ = 7.43 × 10^-10^; OR, 0.283; *P*_c_ = 2.12 × 10^-9^; OR, 0.346 respectively) whereas the frequencies of the GG genotype (*P*_c_ = 7.44 × 10^-10^, OR 3.314) and G allele were significantly higher (Table 3). Further analysis was done in the following four subgroups: AAU with AS (AAU^+^AS^+^), AAU without AS (AAU^+^AS^-^), HLA-B27-positive AAU (AAU^+^B27^+^), and HLA-B27-negative AAU (AAU^+^B27^-^). Our data revealed that the associated polymorphisms of rs12569232/*TRAF5* were consistent among the AAU subgroups investigated (Table 4). We failed to find a significant association with rs6540679 or rs10863888 in the AAU group and its subgroups (data not shown).

**Table 3:** **Genotype and allele frequency analysis between ***TRAF5 ***polymorphisms in AAU patients and healthy controls**

**SNP**	**AAU (*n *** **= 450)**	**P**_**c **_**(OR)**	**Control (*n *** **= 1,601)**
rs12569232			
CC	4	NS	20
CG	28	7.43 × 10^-10^ (0.283)	304
GG	418	7.44 × 10^-10^ (3.314)	1,277
C	4.0%	2.12 × 10^-9^ (0.346)	10.7%
G	96.0%		89.3%
rs10863888			
AA	50	NS	170
AG	202	NS	650
GG	196	NS	754
A	33.7%	NS	31.4%
G	66.3%	NS	68.6%
rs6540679			
AA	17	NS	81
AG	171	NS	497
GG	260	NS	982
A	22.9%	NS	21.1%
G	77.1%	NS	78.9%

**Table 4:** **Genotype and allele frequency analysis between rs12569232/*TRAF5 *** **polymorphisms in AAU subgroups and healthy controls**

**AAU**	**Genotype and allele frequency of rs12569232**			
**Subgroups**	**CC**	**CG**	**GG**	**C**
AAU^+^AS^+^(*n* = 209)	3	14	192	4.8%
*P*_c_ (OR)	NS	1.36 × 10^-4^ (0.306)	3.10 × 10^-4^ (2.866)	5.60 × 10^-4^(0.417)
AAU^+^AS^-^(*n* = 241)	1	14	226	3.3%
*P*_c_ (OR)	NS	5.38 × 10^-6^ (0.263)	2.0 × 10^-6^ (3.823)	1.24 × 10^-6^ (0.285)
AAU^+^B27^+^(n = 267)	2	18	247	4.1%
*P*_c_ (OR)	NS	1.12 × 10^-5^ (0.308)	7.85 × 10^-6^ (3.133)	7.48 × 10^-6^ (0.357)
AAU^+^B27^-^(*n* = 65)	1	3	61	3.8%
*P*_c_ (OR)	NS	0.036 (0.206)	NS	0.048 (0.332)

As rs6540679 and rs10863888 did not show a significant association, they are not shown in the subgroups table.

+, having this feature; -, not having this feature.

### *TRAF5* gene implicated in susceptibility to pediatric uveitis

In total, 458 pediatric uveitis patients were genotyped for three *TRAF5* SNPs. The frequencies of the CG genotype and C allele of rs12569232 were significantly decreased in cases compared with normal controls (*P*_c_ = 3.85 × 10^-6^; OR, 0.420; *P*_c_ = 3.0 × 10^-8^; OR, 0.390), whereas the GG genotype (*P*_c_ = 2.41 × 10^-7^; OR, 2.574) and G allele were both increased. As to rs6540679, a decreased frequency of the AA genotype was observed with a *P*_c_ = 5.64 × 10^-5^;OR, 0.163. No association was found for rs10863888 (Table 5). We subsequently compared pediatric uveitis patients with controls on the basis of clinical and laboratory observations. JIA, FFA, ANA, and RF were chosen as our target factors. The results revealed that the frequency of the GG genotype and G allele of rs12569232 was increased in the patient group with microvascular leakage detected by FFA, whereas the CG genotype and C-allele frequencies were decreased compared with healthy controls (Table 6). No association was found for rs12569232 in patients that had a negative FFA examination. FFA status was not associated with rs10863888 and rs6540679. Furthermore, no significant associations were found when patients were subdivided according to their RF or ANA status or whether they had JIA (data not shown).

**Table 5:** **Genotype and allele-frequency analysis of ***TRAF5 ***polymorphisms in pediatric uveitis patients and healthy controls**

**SNP**	**Pediatric uveitis (*n *** **= 458)**	***P***_**c **_**(OR)**	**Control (*n *** **= 1,601)**
rs12569232			
CC	0	0	20
CG	41	3.85 × 10^-6^ (0.420)	304
GG	416	2.41 × 10^-7^ (2.574)	1,277
C	4.5%	3.0 × 10^-8^ (0.390)	10.7%
G	95.5%		89.3%
rs10863888			
AA	47	NS	170
AG	220	NS	650
GG	191	NS	754
A	34.3%	NS	31.4%
G	65.7%	NS	68.6%
rs6540679			
AA	4	5.64 × 10^-5^ (0.163)	81
AG	152	NS	497
GG	295	NS	982
A	17.7%	NS	21.1%
G	82.3%	NS	78.9%

**Table 6:** **Genotype and allele-frequency analysis of rs12569232/***TRAF5 ***polymorphisms in pediatric uveitis-subgroup patients accompanied by FFA-positive patients**

**SNP**	**Subgroup**	**Genotype**	**Pediatric uveitis**	**Controls**	***P***_**c**_	**OR (95% CI)**
rs12569232	FFA + (*n* = 172)	GG	157	1,277	0.003	2.65 (1.542-4.573)
		CG	15	304	0.012	0.408 (0.237-0.702)
		CC	0	20	NS	
		G	329	2,858	7.68 × 10^-3^	2.640 (1.555-4.483)
		C	15	344	7.68 × 10^-3^	0.379 (0.223-0.643)

SNP, single-nucleotide polymorphism; OR, odds ratio; CI, confidence interval; NS, no statistical difference. P_c_, Bonferroni corrected *P* value compared with normal controls. +, having this feature, -, not having this feature.

## Discussion

In the present study, we aimed to determine whether *TRAF5* gene polymorphisms were associated with AAU and pediatric uveitis in a Han Chinese population. The results showed that rs12569232 polymorphisms of the *TRAF5* gene were associated with both AAU and its subgroups (either accompanied by AS or HLA-B27 positive). The same polymorphism was also associated with pediatric uveitis and, most notably, in the group showing retinal vascular leakage. The frequencies of the CG genotype and C allele of rs12569232 in these test groups were much lower than in the healthy control group, whereas the frequencies of the GG genotype and G allele were significantly higher. A significant association with rs6540679 was found in the pediatric uveitis patients, whereas the frequency of the AA genotype was lower in the patient group compared with controls. No association was found with rs10863888 either in AAU and its subgroups or in pediatric uveitis and its subgroups. These data suggest that *TRAF5* gene polymorphisms may increase the risk for AAU and pediatric uveitis. In the latter group, the association increases the risk for the occurrence of retinal vasculitis.

To our knowledge, this is the first report addressing an association between genetic variants of *TRAF5* and uveitis. The similar association of rs12569232 with these two widely differing uveitis entities described in our study may be due to the fact that the eye only has a limited repertoire with which to respond to inflammation and that *TRAF5* is possibly involved in this final common intraocular inflammatory pathway. Our group is currently studying the role of *TRAF5* in other uveitis entities, such as Vogt-Koyanagi-Harada syndrome and Behḉet disease, and data obtained so far confirm the results presented here. As yet, no other studies have shown an association of rs12569232 *TRAF5* polymorphisms with disease.

*TRAF5*, a tumor necrosis factor receptor-associated factor family protein, acts as an activator of the TNF-induced NF-κB signal pathway [25,40]. It is expressed in various organs and peripheral blood [25,41,42]. Overexpression can lead to inflammation and autoimmune disease, and it has also been shown to protect cells from apoptosis [26,40,43-46]. In view of the findings mentioned earlier, we hypothesized that *TRAF5* may also mediate the development of uveitis. The selection of the *TRAF5* SNPs was based on previous studies concerning the association with rheumatoid arthritis (see Potter *et al*. [35], Supplementary Table) [35] and the observed overlap between uveitis and arthritis. We chose rs6540679, rs12569232, and rs10863888 as our target SNPs for *TRAF5*. We also wanted to include rs7514863, an upstream SNP of *TRAF5* shown to be associated with RA [35], but failed to develop a reliable PCR method. Moreover, a TaqMan SNP Genotyping Assay is not yet available for rs7514863.

Our data showed a significant association with polymorphisms of rs6540679 and rs12569232 that were not observed in RA. The reasons for this discrepancy are unclear, but it could point to different roles of *TRAF5* in the pathogenesis of arthritis as compared with intraocular inflammation. We furthermore investigated whether these mutations were related not only to the occurrence of disease but also to the disease activity. This was done by analyzing a large number of laboratory parameters for pediatric uveitis, including ANA, RF, ASO (anti-streptolysin "O"), CRP (C-reactive protein), ANCA (anti-neutrophil cytoplasmic antibodies), and ESR (erythrocyte sedimentation rate). In AAU, we analyzed the HLA-B27 status of the patients [47]. Clinical manifestations were analyzed for pediatric uveitis with JIA (both oligoarthritis and polyarthritis), arthritis deformans, retinal vasculitis (determined by FFA test) [17,48], and the presence of AS was estimated for AAU patients. Positivity rates of the laboratory tests were below 15%, and no associations with *TRAF5* gene polymorphisms could be found (data not shown). As mentioned, we did note an association between rs12569232 and the presence of retinal vascular leakage in pediatric uveitis patients. Why rs12569232 is associated with retinal vasculitis in PU and why AAU patients with this mutation do not have retinal vasculitis is an intriguing observation that deserves further study.

Further study also is needed to correlate the *TRAF5* genetic data with ophthalmic findings, such as the response to the use of steroids, visual outcome, and recurrence rate. This was not yet possible in the current study, because many of our patients are referred to us by other centers all over China, and the exact medical history concerning treatment and further follow-up of patients is often difficult. Furthermore subdivision into several groups would markedly reduce sample size, thereby reducing the statistical power of the analysis. A future prospective multicenter trial would allow sufficient sample size to address this issue.

We made the following efforts to validate the obtained data. First, the sample sizes of patients and normal controls were large enough to ensure an association analysis. We excluded non-Han patients, and healthy controls were obtained from the same geographic regions as the patients to avoid confounding by genetic ancestry. Furthermore, the diagnosis was performed strictly according to the criteria described previously. Additionally, 10% of the total samples were randomly selected to undergo direct sequencing so as to validate the results of genotyping with PCR-RFLP and TaqMan SNP Genotyping Assay.

Despite these efforts, some limitations exist in our study. We compared our pediatric uveitis patients with a large group of normal healthy adults, instead of using age-matched controls. The large size of our ethnically matched control group (*n* = 1,601) ensures that we obtained a good representation of the frequency of *TRAF5* gene polymorphisms in the Han Chinese population. As this study was performed only in Han Chinese, further research should be done in other ethnic populations to confirm the results. Because our study recruited patients visiting a department of ophthalmology, only uveitis patients were included. Whether the same relation exists between the *TRAF5* gene and AS patients without uveitis remains to be studied. We studied only the relation with three *TRAF5* SNPs, and further studies, including other SNPs of *TRAF5*, must be performed. Whether the *TRAF5* polymorphisms described here play a functional role is not yet clear, and further functional and association studies are needed to address this issue. A recent study by our group showed that healthy control carriers of the GG genotype in SNP rs6540679/*TRAF5* had a higher *TRAF5* mRNA expression level and enhanced TNF-α and IL-6 secretion compared with AA and AG carriers (unpublished data). We were not able to find an effect of the various rs12569232 *TRAF5* genotypes on *TRAF5* mRNA expression by stimulated peripheral blood mononuclear cells (data not shown), and we cannot exclude that the association found reflects an association in linkage disequilibrium with the causative locus. It should also be noted that the GG phenotype of rs12569232 is quite common in China and that *TRAF5* is only one gene in a multihit process involving a large number of genes involved in the regulation of an inflammatory immune response, finally leading to the expression of clinical uveitis.

## Conclusion

Our study investigated the association of *TRAF5* with two common forms of uveitis (AAU and pediatric uveitis) and identified rs12569232 polymorphisms were associated with AAU and its subgroups (accompanied by either AS or HLA-B27 positive). The same mutation was also found in pediatric uveitis and significantly in the group showing retinal vascular leakage, which suggests that rs12569232/*TRAF5* may play a role in the development of retinal vasculitis.

## Abbreviations

AAU: Acute anterior uveitis; ANA: Anti-nuclear antibody; AS: Ankylosing spondylitis; FFA: Fundus fluorescein angiography; HWE: Hardy-Weinberg equilibrium; JIA: Juvenile idiopathic arthritis; OR: Odds ratio; PCR-RFLP: Polymerase chain reaction-restriction fragment length polymorphism; RF: Rheumatoid factor; SNP: Single-nucleotide polymorphism; TNF: Tumor necrosis factor; TRAF5: TNF receptor-associated factor 5.

## Competing interests

No conflicting relation exists for any author, and the authors have no proprietary or commercial interest in any materials discussed in this article.

## Authors’ contributions

QX was listed as XQ, LC was listed as CL, JF was listed as FJ, SH was listed as HSP, PY was listed as YPZ, and AK was listed as AK. LW, LB, YL, YZ were listed as WL, BL, LYJ and ZY, respectively. XQ carried out the gene polymorphism studies, including collecting DNA samples, extracting DNA, genotyping and analyzing the data, and also drafted the manuscript. CL participated in the whole direct sequencing and arrangement of clinical data, and partly participated in DNA sample collection and genotyping. FJ carried out uveitis screening protocols, such as ANA, RF, and FFA. HSP participated in the design of PCR primers and statistical analysis. YPZ conceived of the study and participated in its design and coordination. AK helped to revise the manuscript. WL, BL, LYJ, and ZY carried out DNA sample collection, extraction, and patients’ clinical symptoms gathering. All authors read and approved the final manuscript.
